# Efficient full solar spectrum-driven photocatalytic hydrogen production on low bandgap TiO_2_/conjugated polymer nanostructures†

**DOI:** 10.1039/d3ra04049f

**Published:** 2023-08-10

**Authors:** Edith Mawunya Kutorglo, Michael Schwarze, Anh Dung Nguyen, Simon Djoko Tameu, Shahana Huseyinova, Minoo Tasbihi, Oliver Görke, Matthias Primbs, Miroslav Šoóš, Reinhard Schomäcker

**Affiliations:** a Department of Chemistry, Technische Universität Berlin Straße des 17. Juni 124, TC8 Berlin 10623 Germany kutorgle@vscht.cz; b Bioengineering and Advanced Materials Laboratory, Department of Chemical Engineering, University of Chemistry and Technology Prague Prague 166 28 Czech Republic; c University of Santiago de Compostela, Department of Chemistry Avenida do Mestre Mateo 25 Santiago de Compostela 15706 Spain; d Department of Ceramic Materials, Faculty III: Process Sciences, Technische Universität Berlin Berlin 10623 Germany; e The Electrochemical Energy, Catalysis, and Materials Science Laboratory, Department of Chemistry, Chemical Engineering Division, Technische Universität Berlin Berlin 10623 Germany

## Abstract

The development of photocatalysts that can utilize the entire solar spectrum is crucial to achieving efficient solar energy conversion. The utility of the benchmark photocatalyst, TiO_2_, is limited only to the UV region due to its large bandgap. Extending the light harvesting properties across the entire spectrum is paramount to enhancing solar photocatalytic performance. In this work, we developed low bandgap TiO_2_/conjugated polymer nanostructures which exhibit full spectrum activity for efficient H_2_ production. The highly mesoporous structure of the nanostructures together with the photosensitizing properties of the conjugated polymer enabled efficient solar light activity. The mesoporous TiO_2_ nanostructures calcined at 550 °C exhibited a defect-free anatase crystalline phase with traces of brookite and high surface area, resulting in the best performance in hydrogen production (5.34 mmol g^−1^ h^−1^) under sunlight simulation. This value is higher not only in comparison to other TiO_2_-based catalysts but also to other semiconductor materials reported in the literature. Thus, this work provides an effective strategy for the construction of full spectrum active nanostructured catalysts for enhanced solar photocatalytic hydrogen production.

## Abstract 2

The development of photocatalysts that can utilize the entire solar spectrum is crucial to achieving efficient solar energy conversion.

## Graphical abstract

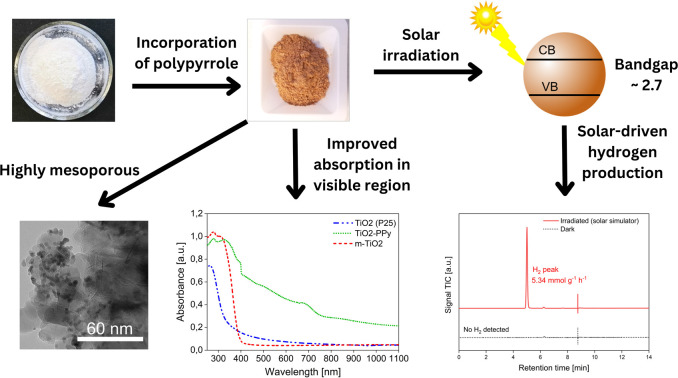

## 1. Introduction

Solar energy-driven hydrogen (H_2_) production from water has been the focus of much current research.^1,2^ This is because sunlight, which is used to activate semiconductor photocatalysts, is a clean and inexhaustible natural resource and the photocatalytic process emits little to no greenhouse gases.^3,4^ Therefore, wide-scale adoption of solar light-driven photocatalytic H_2_ production will be a critical step towards attaining net zero emissions by 2050 (UN sustainable development goals). However, developing full spectrum responsive photocatalysts capable of efficiently utilizing the entire solar spectrum is still a problem for attaining this goal.

TiO_2_ is an effective benchmark photocatalyst due to its non-toxicity, high stability and low cost.^5,6^ Despite its capability to catalyze H_2_ production, the overall efficiency is still far below the minimum requirement for practical applications^7,8^ due to some inherent weaknesses. Firstly, the charge transfer process in TiO_2_ is very slow leading to fast recombination of photoinduced electron–hole pairs.^9,10^ Secondly, TiO_2_ can only be activated under ultraviolet (UV) light irradiation (*λ* < 387 nm) due to its large band gap (∼3.2 eV). To tackle the charge transfer limitation, various sacrificial agents such as ethanol, methanol, and glycerol have been employed to capture the photogenerated holes.^11^ Similarly, metal co-catalysts such as Pd, Ni, Pt, or Cu nanoparticles have been used as electron sinks thereby facilitating efficient electron transfer across the catalyst and preventing electron–hole recombination.^12,13^ Regarding the limitation in light utilization, ultraviolet light constitutes only a small fraction (<5%) of the total solar energy, leading to a low solar-to-hydrogen (STH) conversion efficiency. The STH conversion efficiency describes how much solar energy is converted into chemical energy stored in hydrogen and is calculated using the formula:1
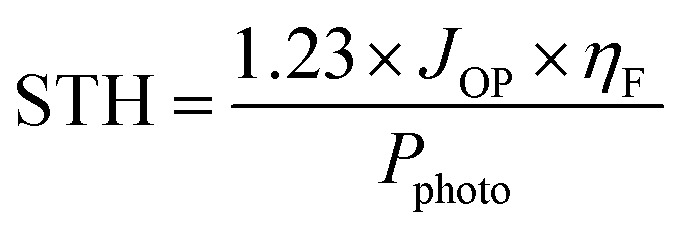
where *η*_F_ is the Faraday efficiency and *P*_photo_ is the solar power. For systems where the amount of hydrogen evolved is measured by gas chromatography or mass spectrometry, the solar to hydrogen efficiency can be evaluated by a more direct method as follows:2
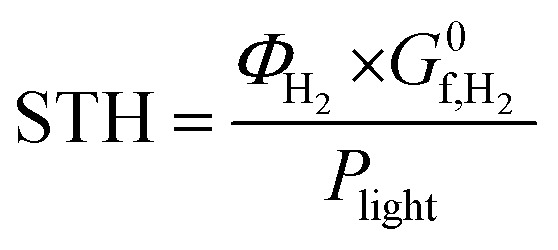
where *Φ*_H_2__ is rate of H_2_ evolution at the illuminated area (mol s^−1^ m^−2^) and 
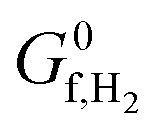
 is the Gibbs free energy of formation of hydrogen (237 kJ mol^−1^).^14^ This means that if TiO_2_ with a band edge of <400 nm is used, the highest theoretical solar-to-hydrogen (STH) conversion efficiency is <2%.^15^ Therefore, there is still a tremendous interest in developing effective solar light-active photocatalysts to improve its photocatalytic performance for practical applications.

Various strategies have been explored to obtain visible light active TiO_2_ including doping, bandgap engineering, metal deposition, surface modifications, composites *etc.*^16–18^ For instance, several researchers^9^ have doped transition metals and non-metals into TiO_2_ structure to enhance the visible light photocatalytic activity. However, doped materials often suffer from thermal or crystal instability and dopant-induced charge recombination, which can decrease the activity.^15,19^ Non-metal doping shows comparably higher promise with nitrogen being the most commonly used dopant.^20,21^

Recently, one of the most studied approaches for enhancing photocatalytic performance is the formation of highly porous nanostructures. Various TiO_2_ nanostructures including nanocrystals,^16^ nanowires,^22^ nanoparticles,^23^ flower-like structures^24^ and hybrid nanoparticles^25^ have been developed, among which, nanostructured catalysts with pores in the nano-to meso range show great promise.^12,26^ This is because their large specific surface areas enhance rapid diffusion of reactants and products and expose more active sites for catalytic reactions thereby enhancing performance.^27,28^ Several researchers have also reported enhanced visible light utilization in highly porous nanostructures because the light bounces back and forth in the cavities allowing more time for absorption and the photoinduced carriers have a higher mobility inhibiting charge recombination.^29,30^ However, till date, harvesting solar light in the entire spectrum (UV to NIR) has still not been fully solved and the practical application of TiO_2_ under solar irradiation remains unrealized. Therefore, it is believed that preparing a highly mesoporous TiO_2_ (m-TiO_2_) catalyst that allows sunlight to diffuse faster through its pores and coupling this catalyst with a visible light-active conjugated polymer such as polypyrrole (PPy)^31^ will be a particularly promising way to enhance solar light response of the photocatalyst for H_2_ production. Conjugated polymers are promising for improving solar energy utilization because of their excellent light harvesting properties in the visible and NIR regions.^32^ Among the conjugated polymers, polypyrrole is the most investigated because of its strong light absorption, low oxidation potential, simple synthesis in aqueous media, good redox properties and low cost.^33^

Herein, we report the synthesis of mesoporous TiO_2_ and TiO_2_-PPy nanostructures by a combined sol–gel and colloidal approach using polyvinyl alcohol as polymeric stabilizer. The as-prepared nanostructures show very high surface areas of greater than 400 m^2^ g^−1^ with a highly mesoporous structure and a very high hydrogen production rate under solar light simulation. This high activity is attributed to a synergistic effect of efficient charge transfer in the highly mesoporous nanostructure and the improved light absorption. A combination of results from *in situ* XRD and H_2_ production measurements, N_2_ adsorption, XRD, XPS, SEM, and UV-vis enabled us to understand how the structural and morphological properties influence photocatalytic activity under simulated sunlight irradiation. The hydrogen production rate obtained under solar simulation is higher compared to previous studies reported in the literature.^21,34,35^ Contrary to other reports about improved H_2_ production performance upon defect introduction and surface area increase,^2,36^ our defect-free anatase mesoporous TiO_2_ with an average surface area performed better than the mesoporous TiO_2_ containing Ti^3+^ and other defects. Thus, this work also provides new viewpoints into the role of textural properties of nanostructures and the presence of defects in enhancing photocatalytic hydrogen production.

## 2. Experimental

### 2.1 Materials

All chemicals were of analytical reagent grade and used without further purification. Hexachloroplatinic(iv) acid hydrate (H_2_PtCl_6_·H_2_O, 8 wt% in H_2_O) was used as Pt precursor. As sacrificial agents for hydrogen production, ethanol (99%, SupraSolv), ascorbic acid (AA, >98%, Sigma Aldrich, Hamburg, Germany), methanol (99.8% purity, VWR chemicals), glycerol, triethanolamine (TEAO, 98% purity, Sigma Aldrich) were used. Titanium tetraisopropoxide (TTIP), poly(vinyl alcohol) PVA (MW 146000–186000 g mol^−1^), pyrrole (Py, 98% purity, Sigma-Aldrich), iron(iii) chloride (FeCl_3_, 97–98% purity, Sigma-Aldrich) and sodium dodecyl sulfate (SDS) were used to synthesize the composites. Platinum ICP standard (Sigma-Aldrich, Hamburg, Germany, 1000 mg L^−1^) was used for calibration of the ICP-OES instrument (Varian ICP-OES 715 ES, radial configuration).

### 2.2 Preparation of m-TiO_2_ and m-TiO_2_/PPy nanostructures

Mesoporous TiO_2_ nanostructures were synthesized by a combined sol–gel and colloidal method using PVA or SDS as stabilizers. In a typical preparation, PVA (100 mg) was dissolved in 20 mL of distilled water at 85 °C. After cooling, the PVA solution was transferred into a 100 mL volumetric flask and the volume was made up to the mark with distilled water. The stabilizer solution was then transferred into a three-neck flask equipped with a condenser and thermometer and heated up to 85 °C. Then 10 mL of TTIP was added under stirring and the pH of the reaction mixture was adjusted to 9 by adding NH_4_OH solution (200 μL). To prepare the TiO_2_-PPy nanostructures, 20 μL of pyrrole monomer was added simultaneously with TTIP to the surfactant solution as in the case of pure m-TiO_2_ preparation. The reaction was left to run for a total of 1 hour resulting in a gelatinous solution. After cooling, the solution was centrifuged and the particles were washed with distilled water several times until the supernatant was clear and the solid was dried at 60 °C for about 12 h. After calcination in air at different temperatures from 200 to 600 °C, the highly crystalline mesoporous TiO_2_ nanostructures were obtained. For the calcination treatments, the temperature was increased by 5 °C min^−1^ from room temperature to the desired temperature and held there for 3 hours.

### 2.3 Deposition of Pt NPs co-catalysts onto the m-TiO_2_ nanostructures

For the deposition of platinum nanoparticles (Pt NPs) on m-TiO_2_ nanostructures, two methods were employed: *in situ* and *ex situ* deposition. *In situ* photodeposition method: the Pt salt precursor H_2_PtCl_6_·H_2_O (60 μL) was added to the reaction mixture containing 120 mL of 10% ethanol solution and 60 mg of m-TiO_2_ nanostructures. The suspension was irradiated for 3 hours using a sunlight simulator (L.O.T. Oriel Quantum Design, Germany) equipped with AM 1.5 filter. After the irradiation step, the dark product was separated by centrifugation, washed 3 times with distilled water, and dried at 60 °C overnight. *Ex situ* reductive deposition method: for comparison, Pt NPs were deposited on m-TiO_2_ nanostructures by the Pt seeded growth method according to a previously published procedure^37^ with slight modifications. The mesoporous TiO_2_ particles were suspended in deionized water containing ascorbic acid and PVA. The mixture was kept under stirring for 10 minutes at 90 °C followed by the addition of the Pt precursor solution. After 10 minutes, a change in colour was observed from cloudy to black, confirming the deposition of Pt onto the TiO_2_ nanostructures. The mesoporous TiO_2_ with Pt NPs deposited were labelled as m-TiO_2_/Pt NPs. The reaction was left to run for another 30 minutes at 90 °C. After the reaction was completed, the resulting particles were washed with distilled water several times by centrifugation and dried at 60 °C overnight.

### 2.4 Materials characterization

The morphology of the photocatalysts was characterized by scanning electron microscopy (SEM). The images were obtained using a JEOL microscope FEG-SEM JSM 6330F operated at 5 kV. Prior to the analysis, the samples were prepared by drop-casting an aqueous suspension of the nanostructures on a Si wafer, followed by drying under ambient conditions. The size distribution profile was determined by individually measuring the size of 200 particles from SEM images. Transmission electron microscopy (HRTEM) images were obtained using a Tecnai FEI G20 instrument operated at 200 kV. Samples were prepared by drop-casting an alcoholic suspension of each particle in a carbon-coated copper grid followed by drying under ambient conditions. The X-ray photoelectron spectroscopy (XPS) analyses were performed using omicron nanotechnology using a monochromatic radiation Al Kα source (*E* = 1486.7 eV) working at 12 kV, *E*_pass_ = 40 eV, with a 0.2 eV energy step in constant analyzer energy (CAE) mode. The BET surface area (*S*_BET_) of the photocatalysts was analyzed using nitrogen adsorption–desorption isotherms with a Micromeritics TriStar 3000 instrument and the data were collected at liquid nitrogen temperature, 77 K. Prior to each measurement, the samples were degassed at 150 °C for at least 2 hours. The specific surface areas were determined using the multipoint BET method using adsorption data in the relative pressure range *P*/*P*_0_ of 0.1–0.3. The total pore volume and average pore size were calculated using the Barrett–Joyner–Halenda (BJH) method^38^ at the relative pressure of 0.996. Powder X-ray diffraction (XRD) patterns of the samples were recorded on an advanced diffractometer (Bruker AXS D8) equipped with a position-sensitive detector (PSD) and a curved germanium (111) primary monochromator. The radiation used was Cu Ka (*λ*¼ 1.5418 Å) and the diffraction patterns were acquired in the range 2*θ* = 10–80° with a 1° min^−1^ scanning speed. Pt atomic percentages were measured by inductively coupled plasma optical emission spectrometry (ICP-OES) using a Varian ICP-OES 715 ES (radial configuration). The samples were prepared by digesting them in aqua regia under stirring for 24 hours at room temperature. After digestion, samples were diluted with distilled water. The optical absorption spectra of the solid samples were measured at room temperature using a Varian Cary 300 UV vis spectrophotometer.

### 2.5 Photocatalytic activity tests (H_2_ production)

The photocatalytic performance of the m-TiO_2_ and m-TiO_2_-PPy nanostructures was examined by the solar-driven H_2_ production conducted in a 250 mL glass photoreactor with a top irradiation window fitted with quartz glass (Fig. S1A in ESI†). The setup was operated at room temperature (approximately 25 °C). Ethanol was used as a renewable sacrificial agent with Pt NPs as co-catalyst for the tests and all experiments were run under simulated solar light conditions. For a typical test, 20 mg of the photocatalyst was placed into the reactor followed by the addition of 40 mL aqueous solution containing 10 vol% of the sacrificial agent. The reactor was closed with a septum fitted cap, connected to a Schlenk line, and purged with argon for 15–20 minutes to replace the air in the headspace with argon. The reaction mixture was irradiated with a solar simulator equipped with an AM 1.5 filter (from L.O.T. Oriel Quantum Design, Germany) under constant stirring for 6 h. The H_2_ produced at the end of the reaction was determined by gas chromatography. For this analysis, 8 mL of gas sample was collected from the headspace using a syringe that has been purged/cleaned with argon. Two samples of 4 mL each were manually injected into a gas chromatograph (GC Agilent 7890 A) equipped with a thermal conductivity detector (TCD). The amount of H_2_ produced in mmol was calculated as follows:
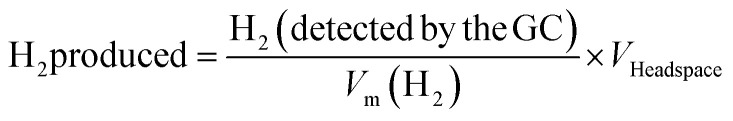
where the *V*_m_ (H_2_) is the molar volume of hydrogen = 24.5 L mol^−1^ (at 25 °C) and *V*_Headspace_ is the headspace volume = 210 mL.

The solar to hydrogen conversion efficiency was evaluated by 
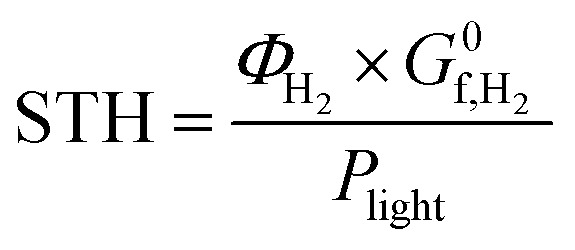
 as detailed in the introduction (eqn (2)).

## 3. Results and discussion

### 3.1 Preparation and structure control of nanostructured catalysts

**Fig. 1:**
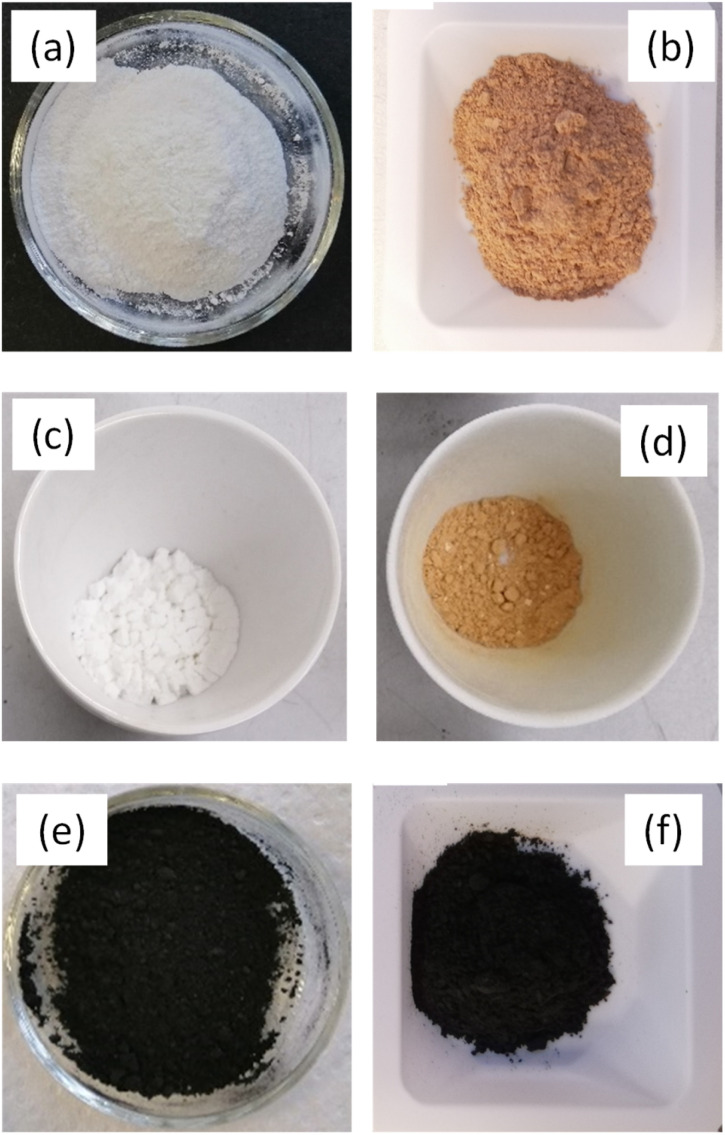
Photos of (a) as prepared m-TiO_2_, (b) as prepared m-TiO_2_/PPy, (c) m-TiO_2_ and (d) m-TiO_2_/PPy calcined in air, (e) m-TiO_2_ and (f) m-TiO_2_/PPy calcined in nitrogen.

The mesoporous TiO_2_ and TiO_2_-PPy nanostructures denoted as m-TiO_2_ and m-TiO_2_-PPy, respectively, were developed by a combined sol–gel and colloidal method to achieve high photocatalytic activity in the entire solar spectrum. The dried m-TiO_2_ nanostructures appeared white after drying (Fig. 1a), whereas an orange-coloured powder was obtained for the m-TiO_2_-PPy nanostructured catalysts (Fig. 1b). The appearance of both m-TiO_2_ and m-TiO_2_-PPy nanostructures remained unchanged after calcination in air (Fig. 1c and d), whereas an obvious colour change from white or orange to black was observed when the powders were calcined under N_2_ atmosphere (Fig. 1e and f). This colour change from white or orange to black is associated with the changes in the lattice structure of TiO_2_ as discussed subsequently. Many researchers have reported on the preparation of defective TiO_2_ (Ti^3+^ sites) which is usually accompanied by a color change from white to black, blue or yellow, among others.^39,40^

**Fig. 2:**
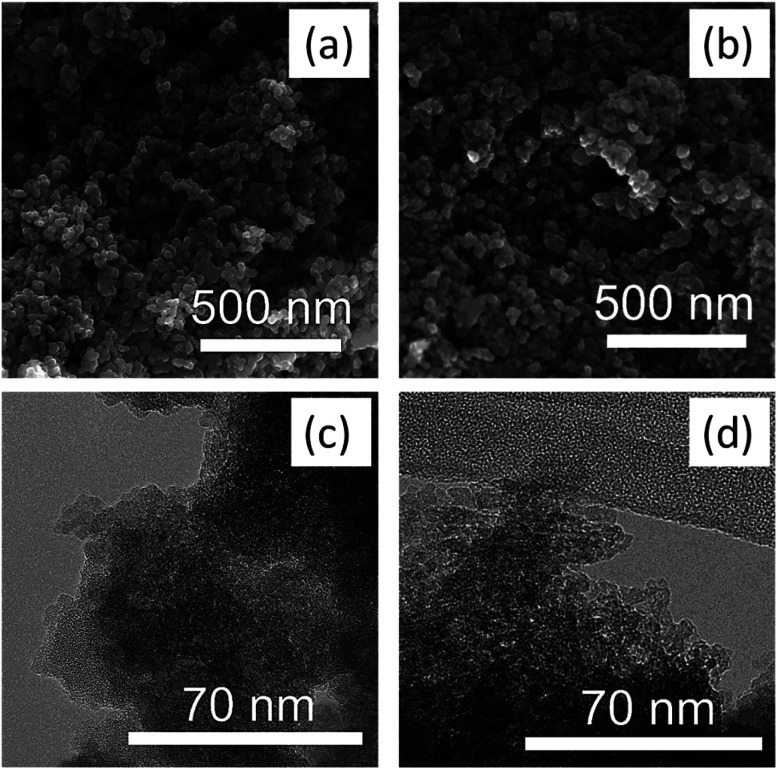
SEM images of (a) m-TiO_2_ (b) m-TiO_2_/PPy nanostructures and the corresponding TEM images of (c) m-TiO_2_ (d) m-TiO_2_/PPy nanostructures.

Electron microscopy images SEM (Fig. 2a) and high-resolution TEM (Fig. 2c) confirmed the highly mesoporous structure of the m-TiO_2_ nanostructures with large surface areas. After incorporation of polypyrrole, the nanostructures were still mesoporous but with a slight increase in the grain sizes as observed in the TEM image (Fig. 2d).

**Fig. 3:**
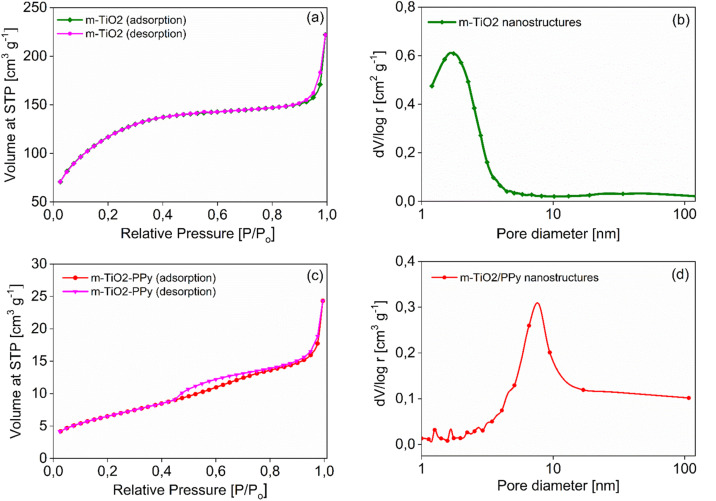
N_2_ sorption isotherms and corresponding pore size distribution curve of m-TiO_2_ (a and b) and m-TiO_2_/PPy nanostructures (c and d).

Fig. 3a and b show N_2_ adsorption data of the m-TiO_2_ nanostructures, showing an average pore diameter of 1.7 nm in the BJH pore size distribution, suggesting the mesoporous nature of the material. These results match well with the SEM and TEM results. The increase in average pore size from 1.7 nm to 7.6 nm after incorporation of PPy can further be seen in Fig. 2d. The increase in average pore size of the m-TiO_2_-PPy can be attributed to the deposition of a layer of PPy around the TiO_2_ structures which also corresponded with a decrease in surface area from 411 m^2^ g^−1^ to 363 m^2^ g^−1^. Investigation of the influence of varying stabilizer amounts (1–5 wt%) on the porous properties of m-TiO_2_ nanostructures revealed that a PVA concentration of 1 wt% was adequate to obtain the highest surface area and a further increase in the stabilizer amount led to a decrease in the surface areas (Fig. S2a in ESI†). Interestingly, when either PVA or SDS was used as a stabilizer in the synthesis of m-TiO_2_, similar BET surface areas and crystal properties were obtained (Fig. S2b†). The hydrolysis temperature also played a key role in the thermal stability and crystalline properties of the resulting m-TiO_2_ during the calcination step. When the hydrolysis step was performed at 85 °C, the resulting m-TiO_2_ showed higher stability to sintering during the calcination process compared to the one synthesized at room temperature (Fig. S3a in ESI†). In addition, the m-TiO_2_ obtained by hydrolysis at room temperature ∼25 °C was amorphous (Fig. S3b,† blue line), whereas the preparation performed at 85 °C yielded the crystalline form of m-TiO_2_. Typically, TiO_2_ materials prepared by traditional sol–gel approach yield amorphous products, which require additional calcination steps to achieve high activity.^28^ Thus, the high crystallinity of the as-prepared m-TiO_2_ (85 °C) without any calcination treatment can be advantageous for sustainably produced catalysts with no high energy input for calcination and no release of poisonous gases. Therefore, 1 wt% stabilizer (PVA) concentration and hydrolysis temperature of 85 °C was used for all the subsequent experiments.

**Fig. 4:**
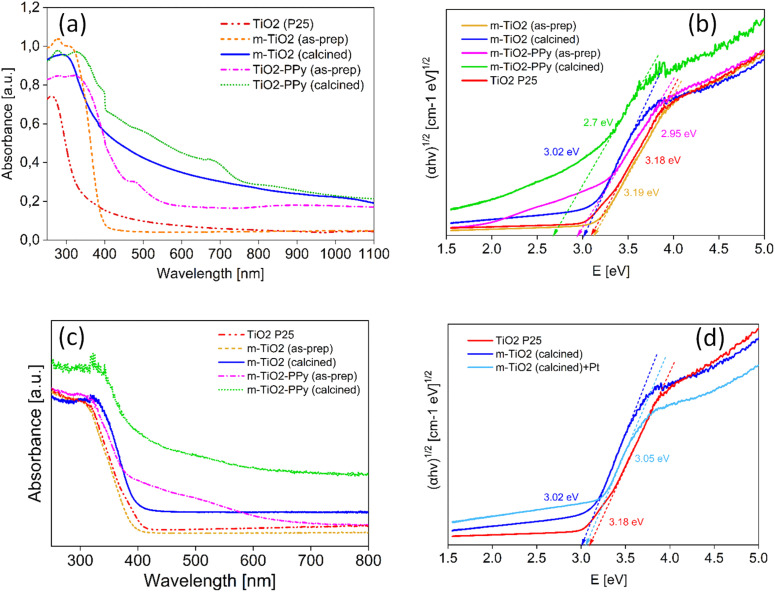
(a) UV-vis spectra of m-TiO_2_ and m-TiO_2_/PPy (in solution) showing enhancement in light absorption in the entire spectrum of light (b) UV-DRS measurements with evaluated bandgap values (c) UV-vis spectra on solid samples and (d) comparison of bandgap of m-TiO_2_ calcines with and without Pt.

To understand the source of the efficient solar light utilization of the m-TiO_2_ and m-TiO_2_-PPy nanostructures, especially in the visible region and NIR regions, absorption spectra were measured on solid materials (Fig. 4). Commercial TiO_2_ P25 shows strong absorption in the UV region absorbing mainly below 350 nm (red curve in Fig. 4a). The spectrum of the m-TiO_2_ nanostructures synthesized in this study showed an absorbance shift to higher wavelengths around 400 nm (orange curves). After calcination of this material, the colour changed to off-white and the absorbance increased in the entire region (blue curve). This implies that the m-TiO_2_ nanostructures can absorb more light in the entire light spectrum when compared to commercial TiO_2_. Upon the introduction of PPy to form the nanostructure with m-TiO_2_, a remarkable increase in absorption is observed in the visible and NIR region (purple curve) in comparison to pure TiO_2_. This enhancement in the light absorption is also consistent with the colour change from white (m-TiO_2_) to orange (m-TiO_2_-PPy) in Fig. 1. After calcination of m-TiO_2_-PPy, the absorption range was mainly between 400 and 800 nm, still confirming good visible light absorption properties. The enhanced absorption can be attributed to the synergistic absorption from the presence of the conjugated polymer PPy^32^ and the highly mesoporous structure of the nanostructures offering more light exposure, which agrees with previous reports.^41,42^ Highly porous structures have also been previously reported to not only enhance efficient diffusion of reactants but also improve visible light absorption.^29,43^

To further confirm the lowering of bandgap and improvement in the light absorption of the materials after the incorporation of PPy, UV-DRS measurements have been conducted and the bandgaps of the materials was estimated (Fig. 4b–d). It was observed that incorporation of PPy resulted in a significant lowering of the bandgap energy of the composites from 3.2 to 2.7 eV. The mesoporous TiO_2_ materials also saw a decrease in bandgap energy from 3.19 to 3.02 eV, further confirming their improved photocatalytic performance.

### 3.2 Photocatalytic hydrogen production tests

**Fig. 5:**
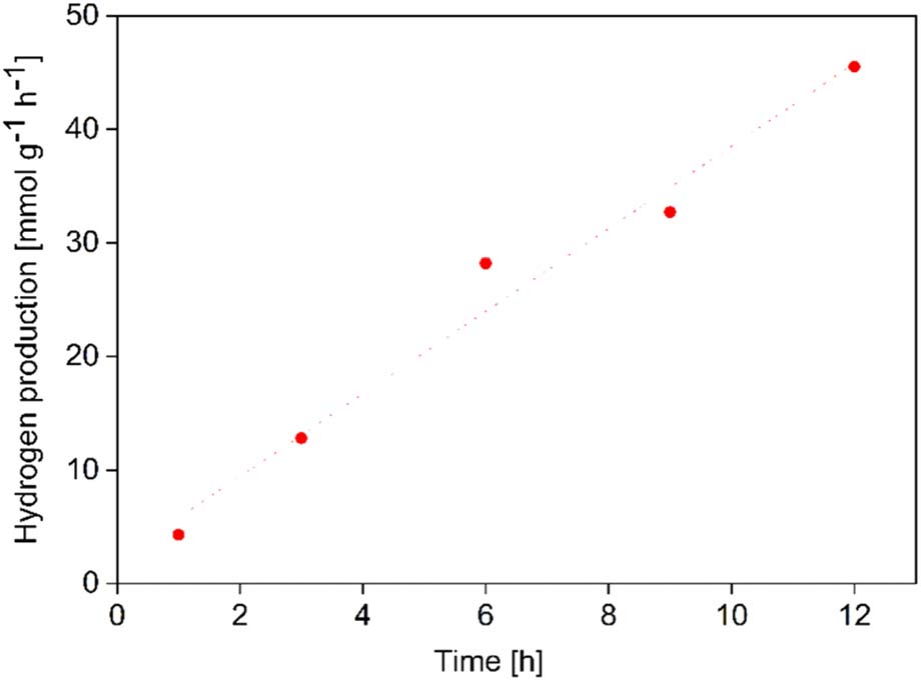
Kinetic studies of hydrogen production on m-TiO_2_ nanostructures over 12 hours using Pt NPs as co-catalyst.

As observed from the light absorption spectra, the m-TiO_2_ and m-TiO_2_-PPy nanostructures predict enhanced photocatalytic activity in the vis and NIR range. Thus, the photocatalytic activity was evaluated for the production of H_2_ under a sunlight simulator using Pt NPs as co-catalyst and ethanol (10%) as the sacrificial agent in a homemade glass photoreactor (Fig. S1A, ESI†). As seen in a typical gas chromatograph of a sample stream (Fig. S1B, ESI†), high amount of H_2_ was detected after just 3 hours of irradiation. Because of the penetration of small amounts of air into the gas syringe used for the sampling, oxygen and nitrogen peaks were observed at low intensity. A steady linear hydrogen production rate was observed for up to 12 hours without any decline (Fig. 5). By performing control experiments with the nanostructured catalysts in the dark without irradiation (Fig. S1B,† black line), no hydrogen was detected. Similarly, irradiation of the reaction in the absence of catalyst yielded no H_2_ confirming that the H_2_ produced comes from the photocatalytic reaction and the m-TiO_2_ and m-TiO_2_-PPy nanostructures show full solar spectrum photocatalytic activity.

The as-prepared m-TiO_2_ nanostructures showed a very good hydrogen evolution rate of 1.36 mmol g^−1^ h^−1^. The hybrid m-TiO_2_-PPy nanostructures exhibited a slightly higher H_2_ production rate of 1.58 mmol g^−1^ h^−1^, which could be due to the enhanced light absorption from the as-prepared PPy hybrid nanostructures, considering that only 0.2 wt% of PPy was incorporated. After the calcination process, the H_2_ evolution activity of the m-TiO_2_-PPy nanostructures increased slightly from 1.58 to 1.95 mmol g^−1^ h^−1^ compared to that of the m-TiO_2,_ whose activity increased about 4 times after calcination in air (1.36 to 5.34 mmol g^−1^ h^−1^). The slower increase in photocatalytic activity of the m-TiO_2_-PPy after the calcination could be due to the inherent instability of conjugated polymers under the calcination conditions (550 °C, air), which is in line with previous reports.^32,44^ Overall, the m-TiO_2_ calcined at 550 °C for 3 hours in air exhibited the highest photocatalytic H_2_ production rate of 5.34 mmol g^−1^ h^−1^ under sunlight simulated conditions using ethanol as a sacrificial agent and Pt NPs as co-catalyst. This value is very high compared to other TiO_2_-based materials reported so far for solar-driven H_2_ production.^7,17,45^

### 3.3 Structure–property relationships

**Fig. 6:**
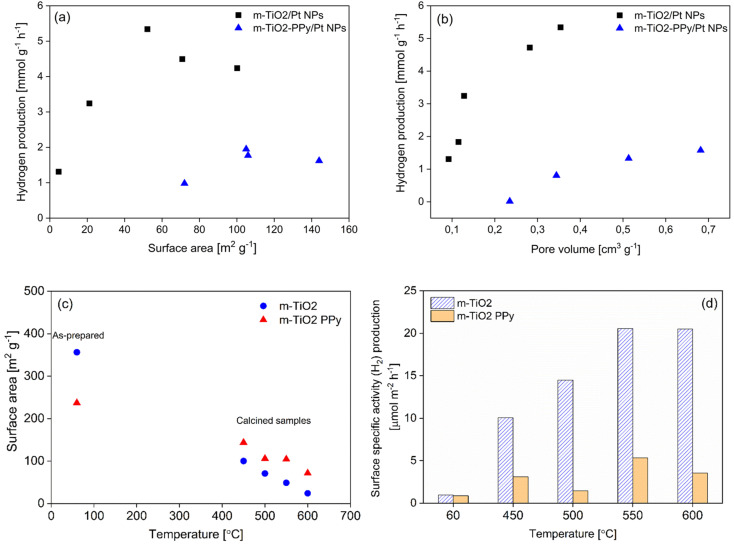
Relationship between (a) BET surface area and hydrogen production, (b) pore volume and hydrogen production, (c) calcination temperature and surface area, and (d) surface-specific activity *vs.* calcination temperature (PPy = 0.2 wt%).

To better understand the factors influencing the activity of the materials, we investigated how the nanostructure properties affect the hydrogen production rate. Fig. 6 shows the comparative photocatalytic hydrogen production using nanostructures of different textural properties to understand the influence of the surface area and pore volume on H_2_ production. Detailed description of the process for tuning of material properties for a series of m-TiO_2_ and m-TiO_2_-PPy samples and subsequent H_2_ production analysis has been given in the Text S1 in ESI.†

**Fig. 7:**
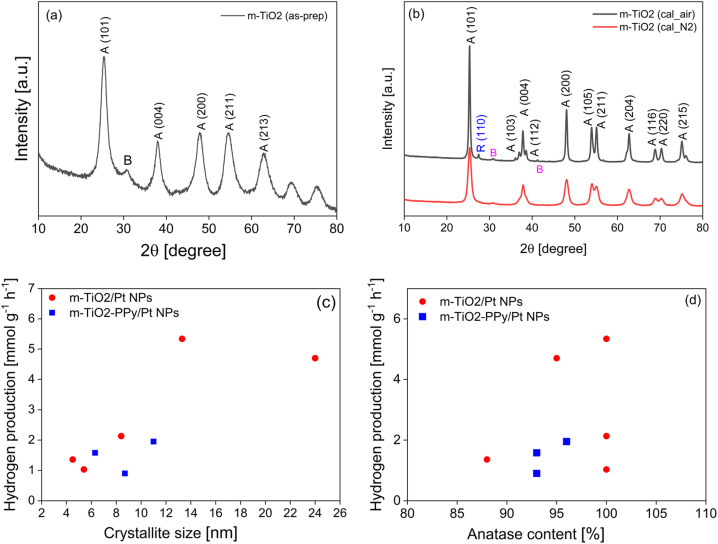
XRD spectra of (a) as-prepared m-TiO_2_ and (b) m-TiO_2_ after calcination under air and N_2_. Relationship between (c) crystallite size and H_2_ production and (d) anatase content and H_2_ production.

No strong correlation was observed between high specific surface areas in m^2^ g^−1^ calculated based on the amount of catalyst used (Fig. 6a), but the hydrogen production increased with increasing pore volumes (Fig. 6b). This could be due to enhanced mass transfer at higher pore volumes. Although calcination treatment of catalysts has been reported to result in crystallization, which further leads to enhanced photocatalytic activity, calcination is also known to induce sintering and aggregation of the catalyst particles which can compromise the photocatalytic activity.^46^ Evaluation of the effect of temperature on the surface area revealed a linear decrease in surface areas with calcination temperature (Fig. 6c). Nevertheless, the surface-specific activity in μmol m^−2^ h^−1^ increased with increasing calcination temperature with the maximum activity at 550 °C for both m-TiO_2_ and m-TiO_2_-PPy nanostructures (Fig. 6d). Comparison of the H_2_ evolution rate for m-TiO_2_/Pt NPs and m-TiO_2_-PPy/Pt NPs with similar pore volume revealed that m-TiO_2_ shows better performance. However, the TiO_2_-PPy has better absorption for the whole solar spectrum. This observation points to the fact that neither the pore volume nor light absorption properties alone control the photocatalytic performance. The crystalline properties also play a crucial role as seen in Fig. 7c and d that the crystalline properties (anatase content and crystallite size) of the m-TiO_2_-PPy are lower compared to that of the pure m-TiO_2_. Overall, these results suggest that having either a high specific surface area or pore volume is not the most critical requirement for enhancing the catalytic activity. Rather, other factors contribute to the hydrogen production such as a more appropriate phase structure and thus, a balance between the porous properties (pore volume), light absorption, and optimal crystallite sizes is required for optimal performance.

To understand the textural changes occurring due to the thermal treatments, the full N_2_ adsorption–desorption isotherms were measured for three conditions where the main changes in the photocatalytic activity occurred (the as-prepared m-TiO_2_, m-TiO_2_ calcined at 550 °C and m-TiO_2_ calcined at 600 °C). The as-prepared m-TiO_2_ shows a type IV isotherm with a characteristic hysteresis loop, which confirms the mesoporous structure (Fig. S5, ESI†). In addition, the isotherm also exhibits high adsorption at low relative pressure, indicating the presence of very small mesopores. In the relative pressure (*P*/*P*_0_) range of 0.9 to 1.0, the materials exhibited very high adsorption, which also points to the simultaneous presence of interparticle macropores. This material exhibited a very high surface area and pore volume of up to 363 m^2^ g^−1^ and 0.34 cm^3^ g^−1^, respectively. Pore size distributions were analyzed using the BJH method from the desorption branch of the isotherm (Fig. S5b, d and f†). After the calcination step, the surface areas decreased to 49 m^2^ g^−1^ and 24 m^2^ g^−1^ for the samples calcined at 550 °C and 600 °C, respectively. Compared to the pore size distribution of the as-prepared m-TiO_2_ with the average pore size of 1.7 nm (Fig. S6b†), the calcined materials (Fig. S6d and f†) exhibited a shift to a broader pore size distribution and larger average pore sizes of 15 nm at 550 °C and >100 nm at 600 °C due to sintering of the catalyst particles. Therefore, the optimum calcination temperature for these TiO_2_ materials was 550 °C which was used in all subsequent experiments. The reduction in the porous properties of the catalyst materials at higher temperatures due to the sintering of the particles has been reported by other researchers.^47^ As presented in Table S1 in ESI,† the catalysts with optimum photocatalytic H_2_ production activity exhibited medium surface areas of about 45–52 m^2^ g^−1^ when compared to the as-prepared materials with surface areas as high as 411 m^2^ g^−1^. Based on these results, we concluded that having extremely high surface area alone is insufficient to control the photocatalytic activity, despite exposing more catalytic active sites.

#### 3.3.1 Catalyst crystalline and surface properties and their influence on H_2_ production

As noted that the high photocatalytic activity of the nanostructures are not only dependent on high surface areas, we investigated the role that the crystalline property of the materials play in the hydrogen production performance. The m-TiO_2_ nanostructures calcined at 550 °C for 3 hours showed the highest H_2_ production rate (5.34 mmol g^−1^ h^−1^), thus this sample was investigated in more detail. To study the reason for the phase and colour changes after the calcination and their influence on H_2_ production, we first analyzed the materials by X-ray diffraction (XRD) and made a correlation between the crystal phase composition and H_2_ production. XRD diffractograms revealed that anatase was the dominating phase and weak peaks of brookite were present. The as-prepared m-TiO_2_ dried at 60 °C was crystalline and composed of a mixture of anatase (88%) and brookite (12%) phases (Fig. 7a). The average crystallite size estimated using the Debye–Scherrer equation was 4.5 nm, which confirmed the presence of nanocrystals of TiO_2_ (Fig. 7a and Table 1). After calcination, the diffraction peaks became narrower and sharper due to an increase in the average crystallite size and improvement of the crystallinity of anatase TiO_2_.^47^ A sharp peak at 2*θ* = 25.3° corresponds to the 101 plane diffraction of anatase TiO_2_ (Fig. 7b) together with other peaks associated with the anatase phase of TiO_2_ (JCPDS powder diffraction file 00-021-1272) were present in all calcined samples. An additional peak at 33.74° can be attributed to the brookite phase of TiO_2_ (JCPDS file no. 291360).

**Table 1:** Crystalline parameters of TiO_2_ before and after calcination^a^

Catalyst	Anatase (%)	Rutile (%)	Brookite (%)	Crystallite size (nm)	BET surface (m^2^ g^−1^)	H_2_ production^b^ (mmol g^−1^ h^−1^)
1. m-TiO_2_ as-prepared	88	—	12	4.5	363	1.36
2. m-TiO_2_ calcined_air	100	-	<1	13.3	42	5.34
3. m-TiO_2_ calcined_N_2_	100	—	<1	8.4	123	2.13
4. m-TiO_2_/PPy as-prepared	93	—	7	6.3	240	1.58
5. m-TiO_2_/PPy calcined_air	96	—	4	11	105	1.95
6. m-TiO_2_/PPy calcined_N_2_	93	—	7	8.7	110	0.90
7. m-TiO_2_ 2 as-prepared	100	—	<1	5.4	362	1.03
8. m-TiO_2_ 2 calcined_air	95	3	2	24	49	4.7

a Calcinations were performed at 550 °C in air or N_2_ for 3 hours.

b The H_2_ production (last column) was performed using Pt NPs as co-catalyst.

The m-TiO_2_ in anatase phase with traces of brookite (<1%) was obtained after calcination in both air and nitrogen. However, the sample calcined in air for 3 hours exhibited a higher H_2_ production rate of 5.34 mmol g^−1^ h^−1^ (Table 1, entry 2) compared to the one calcined under nitrogen atmosphere which was 2.13 mmol g^−1^ h^−1^ (Table 1, entry 3). Similar observations were made in the case of the TiO_2_/PPy composites (Table 1, entries 4–6). Transition between the different TiO_2_ phases has previously been reported after calcination of TiO_2_ at temperatures exceeding 400 °C or 600 °C.^48,49^ In addition, the crystallite size of all the catalysts increased after the calcination. The cross-section of the catalyst particles became larger due to the sintering as also confirmed by the SEM images (Fig. S6 in ESI†). These results together with the series of materials presented in ESI (Table S1†) suggest that the calcination temperature and atmosphere have a strong influence on the crystalline properties and subsequently the photocatalytic activity. Because the surface area of the best material was lower than that of the material calcined under N_2_ whereas both materials are composed of 100% anatase, we investigated the influence of the crystallite size and anatase content on H_2_ production. When plotting the relationship between crystallite size and hydrogen production (Fig. 7c) and it was observed that the photocatalytic activity increased with increasing crystallite size up to 13.3 nm for the m-TiO_2_ materials whereas in the case of the composites, not so obvious changes were observed. The increase in H_2_ production with increasing crystallite size could be due to the suppression of electron–hole recombination. Akinobu Miyoshi *et al.*^45^ have emphasized that larger crystallite sizes mean efficient spatial separation of the redox sites which suppresses electron–hole recombination and enhances photocatalytic activity. The decrease at crystallite size of 24.3 nm could point to the fact that there is a crystal size limit at which the electron transfer is effective and above which the activity decreases or other factors such as the surface area and the type of crystal phase also influenced the activity. The highly crystalline nature of the catalysts containing a mixture of both anatase and traces of brookite phases can also make it possible for interphase charge transfer, which could contribute to the inhibition of charge recombination.^11,50^ Ultimately, a balance between the improved light absorption, crystalline phase structure and an average specific surface area appears to be the optimal conditions for an effective visible light active TiO_2_ photocatalyst for H_2_ production. Evaluating the effect of anatase composition on the photocatalytic activity revealed at first glance that the activity increases with increasing anatase composition (Fig. 7d). However, comparing the three samples with 100% anatase (Table 1, entries 2, 3 and 7), we can say that the anatase composition is only one of the contributing factors that account for the activity.

#### 3.3.2 Influence of surface chemistry on performance

**Fig. 8:**
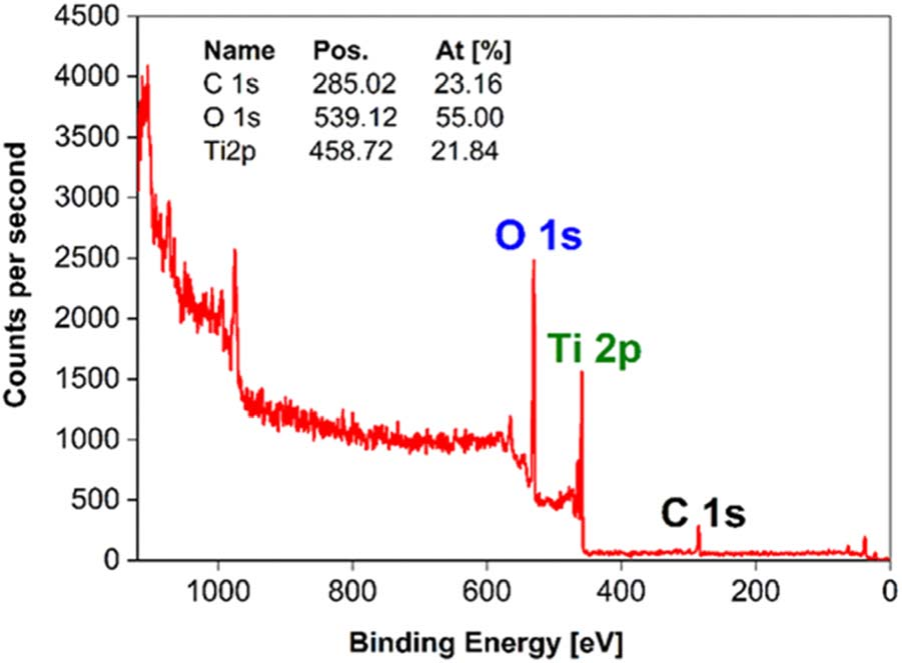
XPS survey spectrum of the m-TiO_2_ nanostructure surface.

**Fig. 9:**
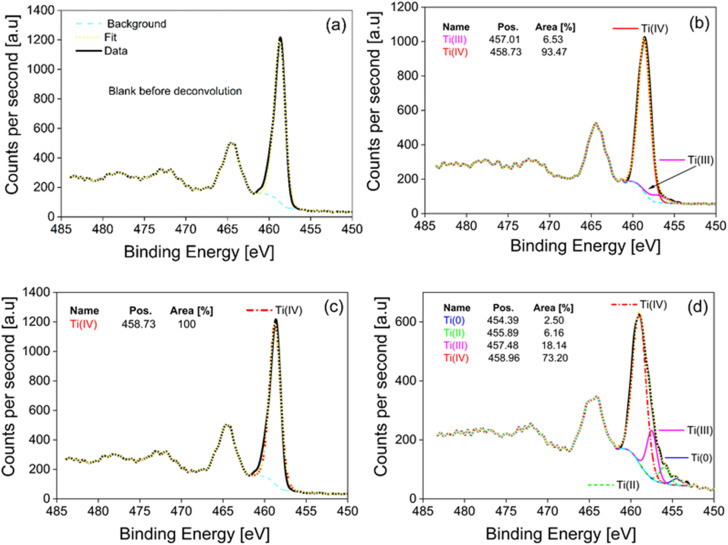
(a) Sample of measurements before deconvolution, (b) m-TiO_2_ as-prepared, (c) m-TiO_2_ calcined in air, and (d) m-TiO_2_ calcined in N_2_.

To investigate the surface chemistry and the chemical states of Ti atoms after the calcination, the samples were characterized by X-ray photoemission spectroscopy (XPS). The XPS survey spectrum (Fig. 8) showed that all the samples contained predominantly Ti 2p (460 eV), O 1s (530 eV), and C 1s (285 eV) peaks. The carbon could be attributed to adventitious carbon,^47,51^ or carbon bonded with the OH group in residual poly(vinyl alcohol) used as a stabilizer during the synthesis. It has been previously reported found that a high number of defects are formed at high temperatures in a reducing environment resulting in the defective m-TiO_2_ structures, which are typically of different colours *i.e.* green, black, yellow, blue *etc.*^52–54^ These defective TiO_2_ materials have been reported to exhibit high photocatalytic H_2_ generation because the Ti^3+^ defects either improved light absorption or acted as co-catalyst centers to facilitate electron transfer.^55–57^ Thus, to understand the difference in the nature of the defect types and their influence on the photocatalytic activity for the different m-TiO_2_, Ti 2p is analyzed together with the fitting and deconvoluted curves (Fig. 9). The corresponding XPS parameters derived from the data are displayed in Table 2. The symmetric Ti 2p_3/2_ and Ti 2p_1/2_ peaks observed at 458.6 eV, 457.37 eV, 455.78 eV, 454.39 eV are attributed to Ti^4+^, Ti^3+^, Ti^2+^ and Ti^0^ respectively of Ti–O bonds. It was observed that the Ti atoms in different samples have different chemical bonding environments based on their thermal treatments and the majority of ionic Ti in all samples was Ti^4+^. The m-TiO_2_ calcined at 550 °C in air showed the presence of only a Ti^4+^ chemical state and exhibited the highest H_2_ production rate (Table 2). The m-TiO_2_ calcined at 550 °C in an N_2_ atmosphere on the other hand showed four chemical states Ti^0^, Ti^2+^, Ti^3+^ and Ti^4+^, with its photocatalytic activity reduced by half. This indicates that the presence of defects could act as recombination sites and thereby reduce the performance of the photocatalyst. This observation is consistent with reports in which Ti^3+^ defects have been reported to act in some cases as recombination centers in TiO_2_ negatively affecting the photocatalytic performance.^5,10,58^ In contrast to other reports about improved performance upon defect introduction,^2,36,59^ we observed that our defect-free anatase mesoporous TiO_2_ performed better than the mesoporous TiO_2_ containing Ti^3+^ and other defects.

**Table 2:** XPS parameters including the surface composition of m-TiO_2_ before and after calcination and the bonding states for peaks deconvoluted for the core level regions of Ti 2p_3/2_ a

Parameter	Ti 2p	O 1s	C 1s	Ti 2p_3/2_	Ti 2p_3/2_	Ti 2p_3/2_	Ti 2p_3/2_	H_2_ production^b^ (mmol g^−1^ h^−1^)
Position	458.72	530.12	285.28	454.39	455.89	457.48	458.96	
				Ti^0^	Ti^2+^	Ti^3+^	Ti^4+^	
1. m-TiO_2_ as-prepared	16.81	51.59	31.61	—	—	6.53	93.47	1.36
2. m-TiO_2_ calcined_air	21.84	55.00	23.16	—	—	—	100	5.34
3. m-TiO_2_ calcined_N_2_	24.93	56.04	19.03	2.50	6.16	18.14	73.20	2.13

a All values are expressed as a percentage (%).

b Hydrogen production tests were conducted with Pt NPs as co-catalyst.

#### 3.3.3 Effect of Pt NP co-catalysts on H_2_ production

**Fig. 10:**
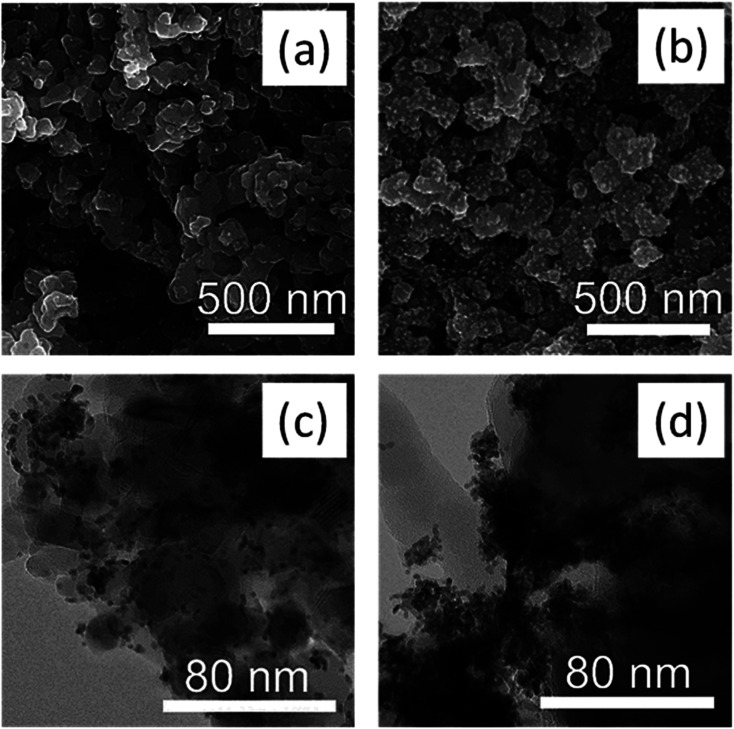
SEM images of mesoporous m-TiO_2_ loaded with Pt NPs by the (a) *in situ* method and (b) *ex situ* method and the corresponding TEM images of *in situ* method (c) and *ex situ* method (d).

Pt NPs are the most active co-catalyst reported for H_2_ production,^5,45^ therefore Pt NPs were immobilized on the surface of m-TiO_2_ by two methods: *in situ* and *ex situ* deposition. The *in situ* approach involved the addition of the Pt precursor of H_2_PtCl_6_·6H_2_O into the reaction mixture containing m-TiO_2_ and aqueous ethanol solution. Upon irradiation with the solar simulator, the Pt species were reduced and deposited onto the surface of m-TiO_2_. The *ex situ* approach involved a seeded chemical method using ascorbic acid as a reducing agent. High-resolution TEM images from the *in situ* photodeposition method (Fig. 10c) showed a good dispersion of the Pt NPs with a very low degree of agglomeration. The Pt NPs are homogenous in size as well as in shape. The Pt NPs from the *ex situ* reductive precipitation method on the other hand showed a rather uneven distribution of the Pt NPs and large aggregates on the surface of the mesoporous TiO_2_ (Fig. 10d).

ICP-OES analysis showed that the *in situ* deposited material had 2.99% Pt whereas the *ex situ* deposited catalyst had 3.28 wt%. EDX was also employed to confirm the presence of Pt NPs deposited on TiO_2_ (Fig. S7 in ESI†). To understand the influence of the Pt amount on the H_2_ production rate, the correlation between the H_2_ production rate and Pt content was analyzed. The as-prepared TiO_2_ materials without Pt co-catalyst showed only moderate H_2_ production rates of 93 μmol g^−1^ h^−1^ (Table 3, entry 1). When Pt was added, the activity increased by about 15 times to 1360 μmol g^−1^ h^−1^ (entry 2). After calcination, the production rate without Pt was 335 μmol g^−1^ h^−1^ (about 3.6 × compared to the as-prepared) because the catalyst particles are crystallized during the calcination process (entry 2). When Pt was loaded onto the calcined sample by the *in situ* approach, the activity increased about 16 times (entry 3). Previous studies have reported that when Pt NPs are loaded onto TiO_2_, they act as an electron sinks, enhancing the separation of photogenerated electron–hole pairs.^50,60^ Moreover, Pt NPs have been reported to act as sensitizers under visible light transferring electrons to the catalyst.^50^ The sample with *ex situ* deposited Pt NPs showed lower activity compared to the *in situ* deposited one despite the higher wt% of Pt in the *ex situ* sample. This could be due to the high degree of aggregation of Pt NPs observed in the *ex situ* deposited catalyst (Fig. 10b and d), which is in line with other studies.^5^ To confirm that the H_2_ produced actually comes from the photocatalyst and not the co-catalyst (Pt NPs), a control experiment using only Pt NPs was conducted. After irradiation of the Pt NPs for about 3 hours, no hydrogen was detected, which confirmed that the H_2_ evolution does not come from the co-catalyst, but rather from the photocatalyst (see Fig. S8 in ESI†). These results indicate that although the presence of Pt NPs is important for improving the catalytic performance, the quantity of Pt present is not the main factor accounting for enhancing the activity, but also the homogeneity of the deposition and size of the particles. Kowalska *et al.*^50^ have reported similar findings that 1 wt% loading of Pt as the optimum and that increasing Pt amount from 1 to 2 wt% did not enhance the performance of the catalyst any further.

**Table 3:** Effect of calcination and Pt loading on H_2_ production rate

Treatments	Catalyst	Pt content (wt%)	H_2_ production (μmol g^−1^ h^−1^)
As-prepared samples	1. m-TiO_2_ as-prepared no Pt	0	93
	2. m-TiO_2_ as-prepared + Pt	2.99	1360
Calcined samples	3. m-TiO_2_ calcined at 550 °C, 3 h in air no Pt	0	335
	4. m-TiO_2_ calcined at 550 °C, 3 h in air + Pt (*in situ*)	2.99	5340
	5. m-TiO_2_ calcined at 550 °C, 3 h in air + Pt (*ex situ*)	3.28	3410

**Fig. 11:**
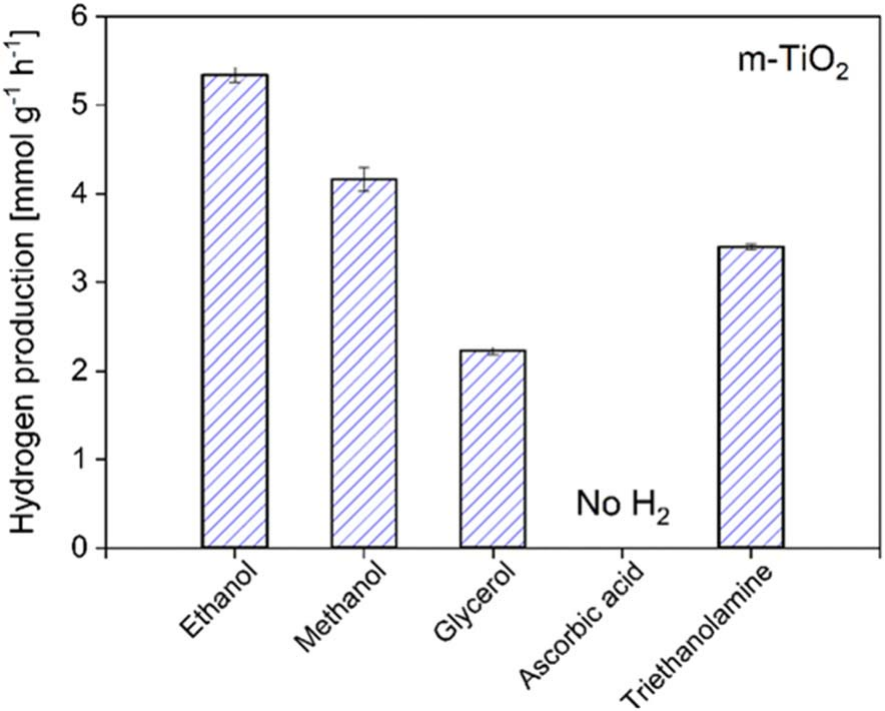
Influence of different sacrificial agents on hydrogen production for m-TiO_2_ nanostructures.

To evaluate the role of the sacrificial agent in the H_2_ production activity as hole scavengers, we tested the best catalyst in pure water with and without Pt co-catalyst and compared the results to that performed with the sacrificial agent (Table 4). We observed that even without any sacrificial agent or Pt added, the m-TiO_2_ was able to produce 28 μmol g^−1^ h^−1^ of H_2_ (entry 1) whereas, with the sacrificial agent and no Pt added, the activity increased to 335 μmol g^−1^ h^−1^ (entry 3). The H_2_ produced from the reaction without a sacrificial agent can only come from water splitting, which demonstrates the capability of the synthesized m-TiO_2_ nanostructures to catalyze overall water splitting but the efficiency needs to be improved. Ideally, the goal is to perform overall water splitting without adding any sacrificial agents, however, to date, only a small number of the developed photocatalysts are capable of driving overall water splitting with good efficiency.^60,61^ Moreover, most of the overall water splitting photocatalysts are structurally complex and their preparation involves multiple steps which can cause environmental pollution. From the point of view of sustainability and life cycle assessment, cheap and simple photocatalysts that utilize renewable sacrificial agents to generate H_2_ are preferable. To evaluate the capability of various renewable sacrificial agents in trapping holes and enhancing the activity, we tested different types of sacrificial agents. As shown in Fig. 11, ethanol was found to produce the highest amount of hydrogen followed by methanol. The differences in the evolution rate of different renewable sacrificial agents could be due to their different capabilities in binding to the catalyst and preventing charge recombination.

**Table 4:** Influence of sacrificial agents on H_2_ production tested with m-TiO_2_ calcined at 550 °C, 3h

Catalyst	Sacrificial agent	Pt	H_2_ production (μmol g^−1^ h^−1^)
1. m-TiO_2_ calcined no Pt, pure water	None	No	28
2. m-TiO_2_ calcined + Pt + pure water	None	Yes	59
3. m-TiO_2_ calcined + no Pt + 10% ethanol	Ethanol	No	335
4. m-TiO_2_ calcined + Pt + 10% ethanol	Ethanol	Yes	5340
5. m-TiO_2_ calcined + Pt + 10% methanol	Methanol	Yes	4163

With more focus on sustainable production and processes nowadays, the simple and low-cost photocatalysts which catalyze HER in the presence of renewable sacrificial agents such as methanol, ethanol, and glycerol, are promising alternatives. These agents aid in enhancing photocatalytic efficiencies by trapping photo-induced holes and thereby prolonging the lifetime of charge carriers to be used in water splitting.^1,11^ Moreover, they can be advantageous over the overall water splitting systems because an additional step would be required as a future challenge for the separation of an explosive O_2_/H_2_ mixture from overall water splitting, which would be unnecessary in the case of sacrificial HER.

Based on all the above analyses, it was demonstrated that there was no hydrogen detected when the developed nanostructured catalysts were placed in the reaction system in the dark. The hydrogen signal was only detected when the nanostructures were irradiated under a solar simulator. This demonstrates the solar activity of the prepared nanostructures, compared to conventional TiO_2_ which shows no vis-NIR activity due to the large bandgap. The best material showed a high H_2_ production rate (5.34 mmol g^−1^ h^−1^), this value is higher for sunlight-driven H_2_ production compared to other semiconductor materials reported in the literature. More effort is however needed to improve the STH efficiency.

Contrary to previous reports^2,30,62^ that the introduction of defects enhances photocatalytic activity, we observed that the presence of these defect states rather compromised the activity, and the defect-free pure anatase phase exhibited the highest activity. The high full solar spectrum activity of the nanostructures can be attributed to several factors:

Both the m-TiO_2_ and m-TiO_2_/PPy nanostructures have a high specific surface area, which can offer more active sites for light absorption and efficient diffusion, thereby improving the photocatalytic properties. Incorporation of the PPy which has excellent absorption properties enhanced the absorption properties in the entire UV-vis spectrum improved the solar light absorption of the nanostructures. And finally, deposition of well dispersed co-catalyst particles onto the surface of the m-TiO_2_ nanostructures can ensure efficient separation of photogenerated charge carriers. The calcination conditions also strongly influence the crystalline properties of the nanostructures and subsequently the photocatalytic activity (Table 5).

**Table 5:** Comparison of solar to hydrogen efficiencies of developed catalysts in the literature

Catalyst system	Co-catalyst	STH conversion efficiency (%)	Ref.
1. Al-doped SrTiO_3_ (SrTiO_3_ : Al)	Rh/Cr_2_O_3_, CoOOH (impregnation)	0.51	62
2. Y_2_Ti_2_O_5_S_2_	Ru/Cr_2_O_3_, IrO_2_	0.007	63
3. B-doped, N-deficient C_3_N_4_	Pt, Co(OH)_2_	1.16	64
4. Ta_3_N_5_	Rh/Cr_2_O_3_	0.014	65
5. SrTiO_3_ : Al	Rh/Cr_2_O_3_, CoOOH	0.65	66
6. m-TiO_2_ and m-TiO_2_/PPy	Pt	0.60	This work

It is an indisputable fact that high surface areas can provide more exposed sites for surface reactions whereas large pore size can shorten diffusion paths to enhance the reaction kinetics. Nevertheless, detailed structure–function investigation from H_2_ production, XRD, BET and XPS results confirmed that high surface area alone is not sufficient to enhance the photocatalytic activity. Rather, a balance of crystalline phase structure, good dispersity of Pt NPs on the surface of the m-TiO_2_, good light absorption properties, and pore architecture (high mesoporosity) are responsible for the high performance.

## 4. Conclusions

We have successfully prepared new mesoporous TiO_2_ and m-TiO_2-_PPy nanostructures with full solar spectrum activity using a simple combination of colloidal and sol–gel method. The high solar photocatalytic activity is attributed to the synergistic effect of the highly mesoporous structure coupled with enhanced light absorption. The as-prepared nanostructured catalysts even prior to calcination treatment showed high crystallinity with very high surface areas up to 400 m^2^ g^−1^ and a reasonably good hydrogen production rate of 1.36 mmol g^−1^ h^−1^ which increased to 5.34 mmol g^−1^ h^−1^ after calcination. This presents a good opportunity for sustainably producing catalysts with no need for high energy input for calcination and its consequent release of poisonous gases. Based on the understanding obtained from this work, the presented approach provides an effective strategy for the design of effective mesoporous TiO_2_-based nanostructured catalysts. Beyond their application in hydrogen production, these catalysts are being tested in CO_2_ reduction to ethane, methane and carbon monoxide confirming their use in a wide range of fields such as fuel cells, and photovoltaics.

## Conflicts of interest

The authors declare that they have no known competing financial interests or personal relationships that could have appeared to influence the work reported in this paper.

## Supplementary Material

RA-013-D3RA04049F-s001
