# Supplementary material for: Efficient full solar spectrum-driven photocatalytic hydrogen production on low bandgap TiO2/conjugated polymer nanostructures
Source: RSC Adv. 2023 Aug 10;13(34):24038–52. doi: 10.1039/d3ra04049f (PMC10414019; doi:10.1039/d3ra04049f)
Supplement: RA-013-D3RA04049F-s001 [file RA-013-D3RA04049F-s001.pdf]

Electronic Supplementary Material (ESI) for RSC Advances.
This journal is © The Royal Society of Chemistry 2023

Supporting Information

Efficient full solar spectrum-driven photocatalytic hydrogen production on low bandgap TiO_2/conjugated polymer nanostructures.

Edith Mawunya Kutorglo[1, 2, *], Michael Schwarze[1], Anh Dung Nguyen[1], Simon Tameu Djoko[1], Shahana Huseyinova[1,3], Minoo Tasbihi[1], Oliver Görke[4], Matthias Primbs[5], Miroslav Šoóš[2], Reinhard Schomäcker[1]

[1]Department of Chemistry, Technische Universität Berlin, Straße des 17. Juni 124, TC8, 10623 Berlin, Germany.

[2]Bioengineering and Advanced Materials Laboratory, Department of Chemical Engineering, University of Chemistry and Technology Prague, 166 28 Prague, Czech Republic.

[3]University of Santiago de Compostela, Department of Chemistry, Avenida do Mestre Mateo 25, 15706 Santiago de Compostela, Spain

[4]Department of Ceramic Materials, Faculty III: Process Sciences, Technische Universität Berlin, 10623 Berlin, Germany.

[5]The Electrochemical Energy, Catalysis, and Materials Science Laboratory, Department of Chemistry, Chemical Engineering Division, Technische Universität Berlin, 10623 Berlin, Germany.

Table of Contents

	Content	Page
1.	Figure SI 1. Setup for the H_2 production tests	2
2.	Figure SI 2. Effect of PVA concentration on BET surface area of m-TiO_2 and (b) effect of hydrolysis temperature on the crystalline properties of TiO_2	3
3.	Figure SI 3. Effect of calcination temperature on BET surface area of m-TiO_2 and (b) effect of hydrolysis temperature on the crystalline properties of m-TiO_2	4
4.	Text S1. Selecting optimum calcination conditions	5
5.	Figure SI 4 Influence of calcination temperature on H_2 production and BET surface area of m-TiO_2	6
6.	Figure SI 5 N_2 adsorption-desorption isotherm of TiO_2 before and after calcination	7
7.	Figure SI 6 SEM images showing sintering effect of TiO_2 calcined at different temperatures. Table S1 summarizing the effect of calcination temperature on H_2 production and textural properties.	8
8.	Fig. SI 7 EDX spectra confirming the presence of Pt NPs on m-TiO_2	9
9.	Fig. SI 8 Control experiment using Pt NPs only with irradiation under solar simulation	10

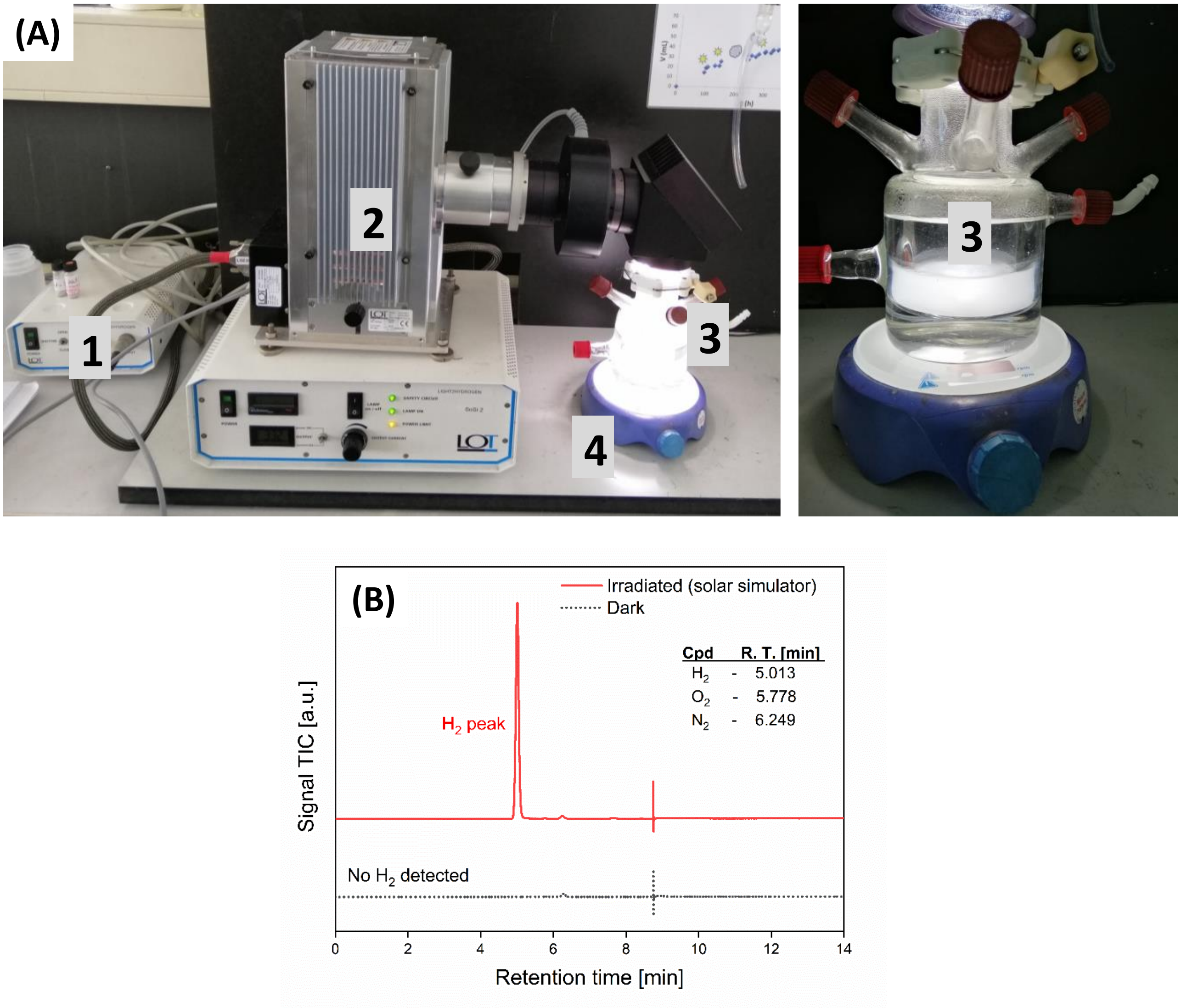

Figure SI 1. A) Setup for the H_2 production tests: (1) Filter, (2) solar simulator (300 W Xe lamp with AM 1.5 filter), (3) top irradiation photoreactor, and (4) magnetic stirrer. B) A gas chromatograph for a collected sample of generated hydrogen stream under a solar simulator.

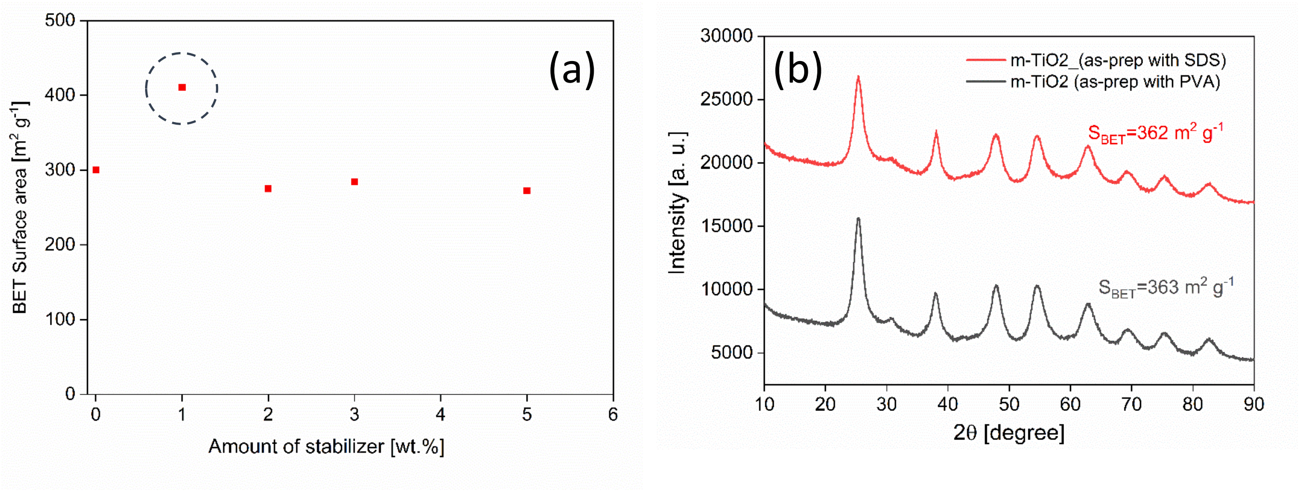

Figure SI 2. (a) Influence of stabilizer amount on BET surface area of m-TiO_2 and (b) XRD pattern for m-TiO_2 prepared with PVA or SDS.

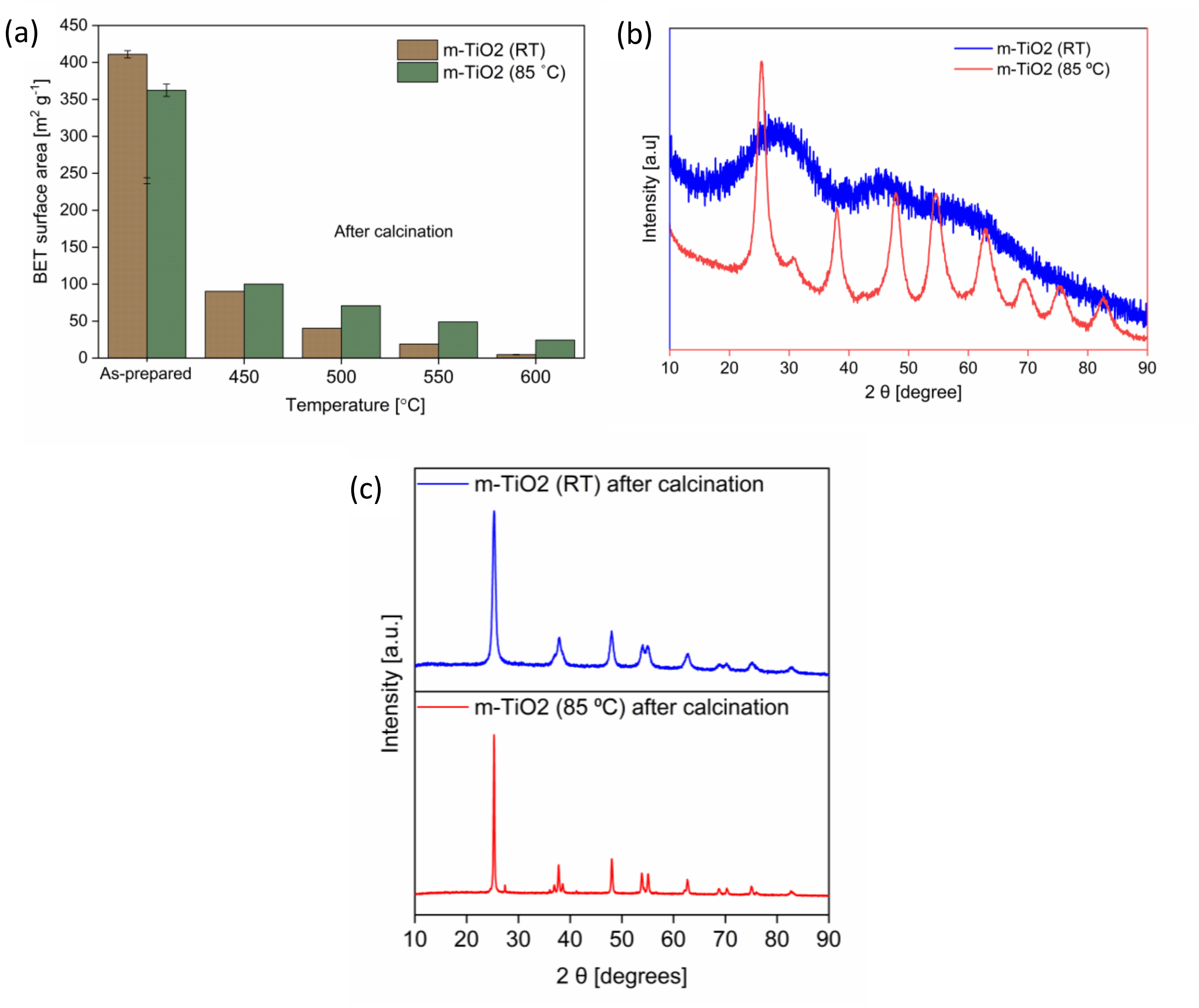

Figure SI 3. (a) Effect of calcination temperature on BET surface area of m-TiO_2 and (b) effect of hydrolysis temperature on the crystalline properties of m-TiO_2 (c) catalysts after calcination

The XRD pattern of the as-prepared catalysts (Figure SI 3 (b)) confirmed that m-TiO_2 prepared at 85 °C was crystalline, whereas TiO_2 prepared at 25 °C was amorphous and was only converted to crystalline upon subsequent calcination treatment (Figure SI 3 (c).

Text S1: Selecting the Optimum calcination conditions

The performance of any given material is dependent on its physicochemical properties, which are determined by the preparation conditions. Although high-temperature treatment in air has been reported to result in crystallization which leads to enhanced photocatalytic activity, calcination is also known to induce sintering and aggregation of the catalyst particles which can compromise the photocatalytic activity[1]. In view of this, we performed calcinations at different temperatures and evaluated the optimal temperature at which the surface area and crystalline properties were preserved and the highest amount of hydrogen was produced. The as-prepared m-TiO_2 exhibited a reasonably good hydrogen evolution activity of 1.36 mmol g^{-1} h^{-1} under simulated sunlight conditions. Increasing the calcination temperature from 200 to 400 °C did not affect the H_2 production compared to the as-prepared material (Figure SI 4, Table SI 1 below). Above this temperature, the rate of hydrogen production increased. Mesoporous TiO_2 nanostructures calcined at 550 °C for 3 h in air showed the highest H_2 production rate. A further increase in the calcination temperature from 550 to 600 °C resulted in a decrease in the hydrogen production rate from 5.34 to 4.24 mmol g^{-1} h^{-1}, which also corresponded to a significant decrease in the surface area from 49 m^2g^{-1} to 24 m^2g^{-1}. This could be attributed to increased interparticle contact and a high degree of sintering of the particles at high temperatures as confirmed by SEM images and a broader pore size distribution (Figure SI 5 and 6, respectively below). These observations are in agreement with other studies which reported a high degree of sintering of catalyst particles at temperatures higher than 600 °C [2]. Miszczak and Pietrzyk [3] have also reported that anatase to rutile phase transition occurs at temperatures above 600 °C which decreases the photocatalytic activity. Overall, these results suggest that heat treatment of m-TiO_2 with temperatures between 450 °C and 550 °C has a positive impact on the photocatalytic H_2 production performance.

Apart from the calcination temperature, the calcination atmosphere also significantly influences the textural properties and photocatalytic activity of the catalysts. When the optimum material m-TiO_2 was calcined under N_2 atmosphere instead of air, the BET surface area increased (See Table SI 1, Entry 9 and 10) but the H_2 production rate decreased to about half. Considering the higher surface area, the decrease in activity could be due to changes in the crystal structure, which was also evident from a color change from white to black (See Scheme 1 (c) in the main text). In addition, the color change also indicates changes in the oxidation states of TiO_2 in an oxygen-deficient environment. Therefore, the surface area is not the main factor determining photocatalytic activity. Evidently, the activity is based on a combination of optimal surface area, absorption, and crystalline properties. In the case of the hybrid m-TiO_2/PPy nanostructures, a similar trend was observed upon calcination with the optimum material obtained after calcination at 550 °C for 3 h in air. Nonetheless, the H_2 production rate of the calcined TiO_2-PPy composite material increased only slightly after calcination (from 1.58 to 1.95 mmol $g^{-1}h^{-1}$) compared to that of the m-TiO_2, whose activity increased about 4 times after calcination in air (1.36 to 5.34 mmol g^{-1} h^{-1}). The lower photocatalytic activity of the composites after the calcination could be due to the instability of PPy under the calcination conditions (550 °C, air), which is in line with previous reports [4]. These results are summarized in Table S1, below. To test whether the degradation of PPy can be prevented, m-TiO_2/PPy was calcined under an N_2 atmosphere. Similar to the observation with pure m-TiO_2, the m-TiO_2/PPy calcined under an N_2 atmosphere turned black in color, and its activity was reduced by half (Table S1, Entries 16 in Supporting Information). In the future, we plan to investigate photosensitizers with higher thermal stability such as porphyrins or metal phthalocyanines.

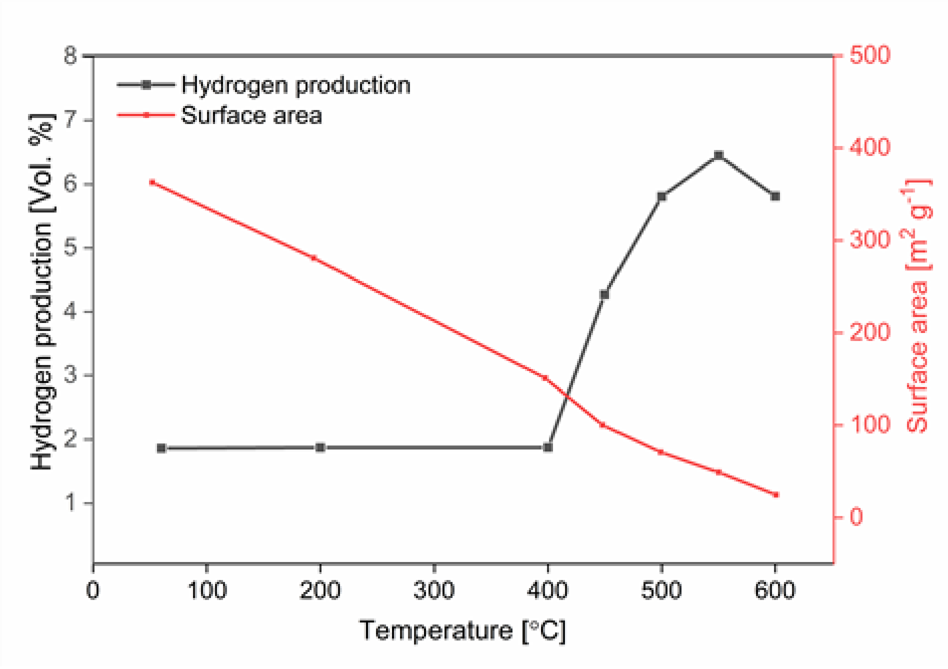

Figure SI 4. Influence of calcination temperature on H_2 production and BET surface area of m-TiO_2.

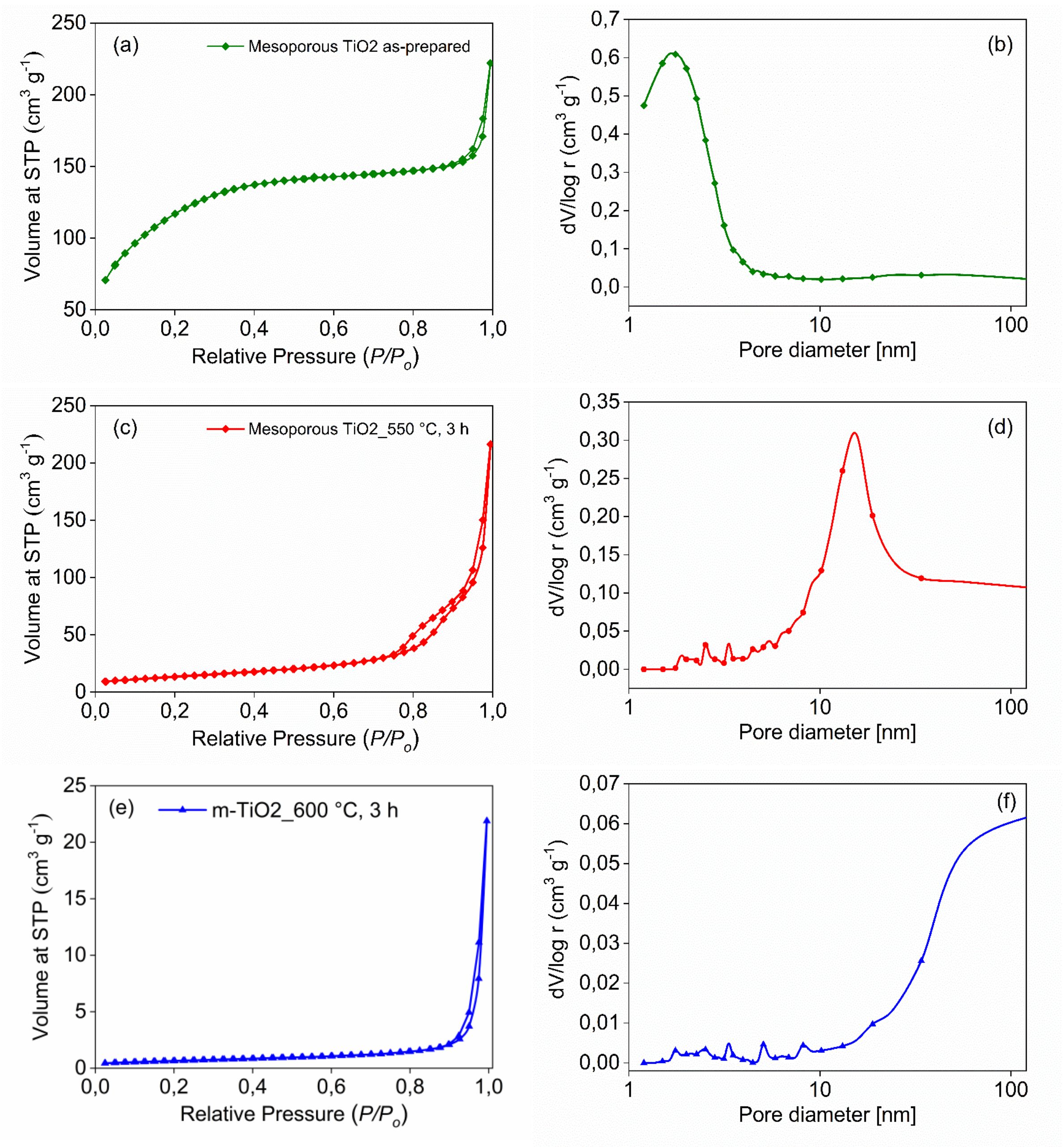

Figure SI 5. N_2 adsorption-desorption isotherm of (a) as-prepared m-TiO_2, (c) m-TiO_2 calcined for 3h at 550 °C, (e) m-TiO_2 calcined for 3h at 600 °C, and their corresponding pore size distributions (b), (d) and (f).

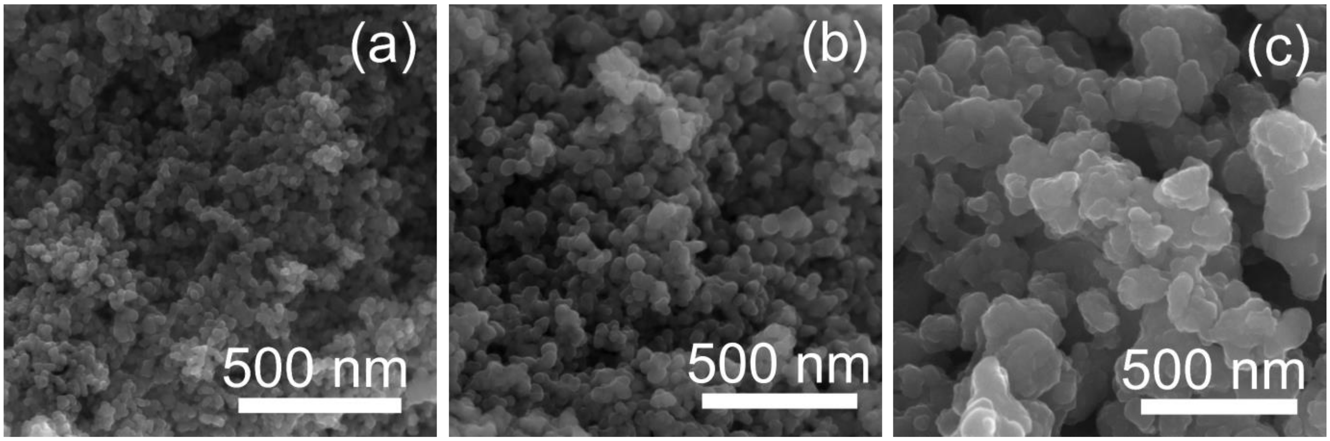

Figure SI 6. Sintering effect of TiO_2 calcined at different temperatures (a) as-prepared, (b) calcined at 550 °C, and (c) calcined at 600 °C.

Table SI 1. Effect of calcination temperature on H_2 production and textural properties, calcination was performed in air in all cases for 3 hours. m-TiO_2 (RT) and m-TiO_2 (HT) refer to mesoporous TiO_2 where hydrolysis was performed at room temperature and 85 °C, respectively.

	Sample	Calcination Temperature [°C]	BET Surface Area [m^2g^{-1}]	H_2 production [mmol g^{-1} h^{-1}]
1.	m-TiO_2 (RT) as-prepared	60	411	1.36
2.	m-TiO_2 (RT)_450	450	90	2.77
3.	m-TiO_2 (RT)_500	500	40	2.89
4.	m-TiO_2 (RT)_550	550	21	3.24
5.	m-TiO_2 (RT)_600	600	5	1.31
6.	m-TiO_2 (HT) as-prepared	60	363	1.36
7.	m-TiO_2 (HT)_450	450	100	4.24
8.	m-TiO_2 (HT)_500	500	71	4.49
9.	m-TiO_2 (HT)_550 in air	550	49	5.34
10.	***m-TiO_2 (HT)_550 under N_2***	***550***	***123***	***2.13***
11.	m-TiO_2 (HT)_600	600	24	4.24
12.	TiO_2-PPy_as-prepared	60	237	1.58
13.	TiO_2-PPy_450	450	144	1.62
14.	TiO_2-PPy_500	500	106	1.77
15.	TiO_2-PPy_550 in air	550	105	1.95
16.	***TiO_2-PPy_550 under N_2***	***550***	***110***	***0.90***
17.	TiO_2-PPy_600	600	72	0.98

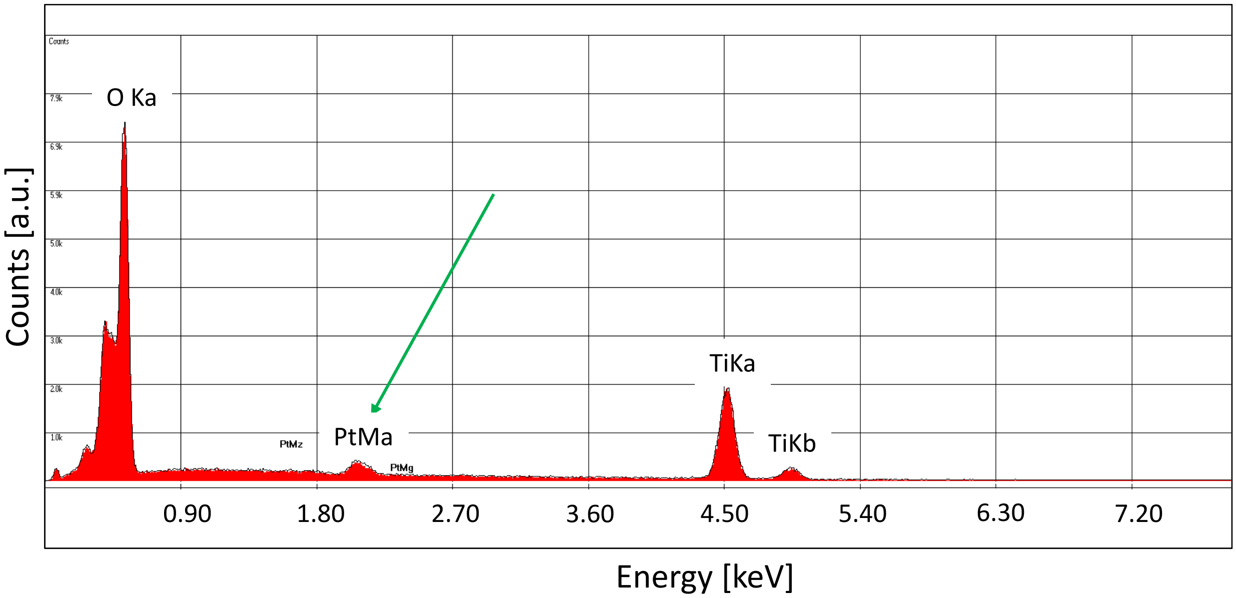

Figure SI 7. EDX analysis confirming the presence of Pt NPs on m-TiO_2.

Setup for the control experiment with Pt NPs only.

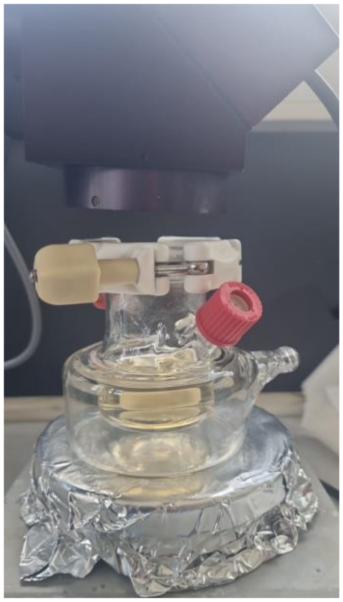

Gas chromatograph after the irradiation step with no hydrogen gas detected.

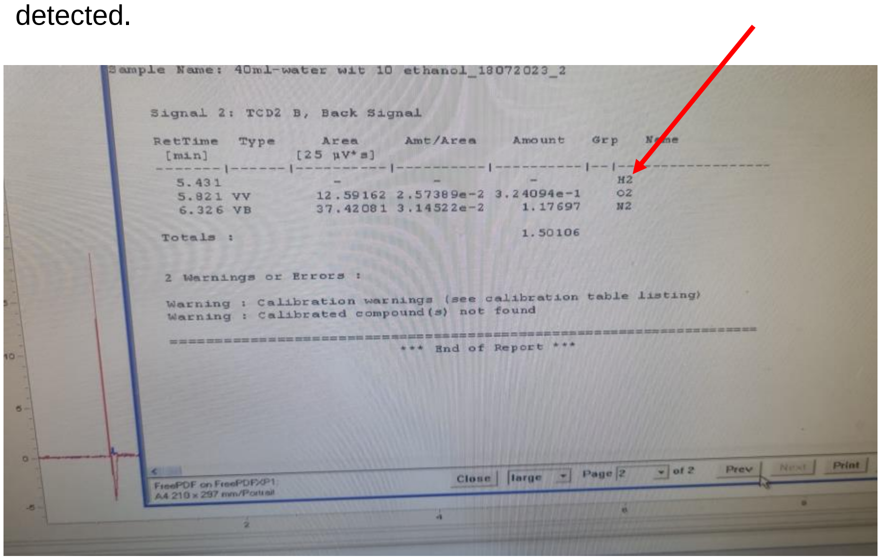

Fig. SI 8 Control experiment using Pt NPs only with irradiation under solar simulation

References

[1] K. Al-Attafi, A. Nattestad, Q. Wu, Y. Ide, Y. Yamauchi, S.X. Dou, J.H. Kim, Chem. Commun. (Camb), 54 (2018) 381-384, doi:10.1039/c7cc07559f.

[2] W. Wang, C.-H. Lu, Y.-R. Ni, J.-B. Song, M.-X. Su, Z.-Z. Xu, Catal. Comm., 22 (2012) 19-23, doi:10.1016/j.catcom.2012.02.011.

[3] Miszczak, S.; Pietrzyk, B., Ceramics International 2015, 41 (6), 7461-7465.

[4] Z. Ahmad, M.A. Choudhary, A. Mehmood, R. Wakeel, T. Akhtar, M.A. Rafiq, Macromol. Res., 24 (2016) 596-601, doi:10.1007/s13233-016-4081-x.
